# Quantitative tissue proteome profile reveals neutrophil degranulation and remodeling of extracellular matrix proteins in early stage gallbladder cancer

**DOI:** 10.3389/fonc.2022.1046974

**Published:** 2023-01-06

**Authors:** Javed Akhtar, Vaishali Jain, Radhika Kansal, Ratna Priya, Puja Sakhuja, Surbhi Goyal, Anil Kumar Agarwal, Vivek Ghose, Ravindra Varma Polisetty, Ravi Sirdeshmukh, Sudeshna Kar, Poonam Gautam

**Affiliations:** ^1^ Laboratory of Molecular Oncology, Indian Council of Medical Research (ICMR) - National Institute of Pathology, New Delhi, India; ^2^ Jamia Hamdard- Institute of Molecular Medicine, Jamia Hamdard, New Delhi, India; ^3^ Department (Nil), Manipal Academy of Higher Education (MAHE), Manipal, India; ^4^ Department of Pathology, Govind Ballabh Pant Institute of Postgraduate Medical Education and Research (GIPMER), New Delhi, India; ^5^ Institute of Bioinformatics, International Tech Park, Bangalore, India; ^6^ Department of Biochemistry, Sri Venkateswara College, University of Delhi, New Delhi, India

**Keywords:** gallbladder cancer, early stage, neutrophil degranulation, tissue proteomics, iTRAQ

## Abstract

Gallbladder cancer (GBC) is an aggressive malignancy of the gastrointestinal tract with a poor prognosis. It is important to understand the molecular processes associated with the pathogenesis of early stage GBC and identify proteins useful for diagnostic and therapeutic strategies. Here, we have carried out an iTRAQ-based quantitative proteomic analysis of tumor tissues from early stage GBC cases (stage I, n=7 and stage II, n=5) and non-tumor controls (n=6) from gallstone disease (GSD). We identified 357 differentially expressed proteins (DEPs) based on ≥ 2 unique peptides and ≥ 2 fold change with p value < 0.05. Pathway analysis using the STRING database showed, ‘neutrophil degranulation’ to be the major upregulated pathway that includes proteins such as MPO, PRTN3, S100A8, MMP9, DEFA1, AZU, and ‘ECM organization’ to be the major downregulated pathway that includes proteins such as COL14A1, COL1A2, COL6A1, COL6A2, COL6A3, BGN, DCN. Western blot and/or IHC analysis confirmed the elevated expression of MPO, PRTN3 and S100A8 in early stage of the disease. Based on the above results, we hypothesize that there is an increased neutrophil infiltration in tumor tissue and neutrophil degranulation leading to degradation of extracellular matrix (ECM) proteins promoting cancer cell invasion in the early stage GBC. Some of the proteins (MPO, MMP9, DEFA1) associated with ‘neutrophil degranulation’ showed the presence of ‘signal sequence’ suggesting their potential as circulatory markers for early detection of GBC. Overall, the study presents a protein dataset associated with early stage GBC.

## 1 Introduction

Gallbladder cancer (GBC) is the fifth most common and aggressive malignancy of the gastrointestinal tract, with a marked geographical variation in its incidence. There are two major groups of high-risk populations for GBC, in Latin America (Chile, US Native Americans, Mexicans) and in Asia (Northern India, Pakistan, Korea, Japan and China) (1, 2). Among the Asian countries, GBC has the highest prevalence and incidence rate in northern and northeast India (1, 3, 4). Gallstone disease (GSD) cases and female population are at high risk for GBC (5). GBC is generally diagnosed at an advanced stage due to its anatomic position and non-specific symptoms. Imaging techniques and the available blood tests (CEA, CA19-9) are generally employed for the diagnosis of GBC, however, the detection of the disease at early stage remains a challenge. The treatment includes extended resection in combination with chemotherapy, radio-therapy and targeted therapy (6).

In early stage GBC (Stage I and II), the tumor is restricted to the gallbladder while in advanced stages (Stages III and IV), the tumor invades beyond the gallbladder serosa to the liver or other nearby structures *via* direct invasion or lymphatic, peritoneal and hematogenous dissemination (7). Application of high throughput approaches to understand the molecular profile of ‘early stage GBC’ is important to identify ‘tumor-associated proteins’ and associated molecular pathways which may be useful as new diagnostic markers and therapeutic targets.

There are several studies on genetic, epigenetic and transcript analysis of tumor tissues and cell lines to understand the molecular changes associated with GBC (8–10). p53 mutation, mitochondrial DNA mutation, cyclooxygenase-2 (COX2) overexpression, methylation of tumor suppressor gene (TSG) promoters and/or KRAS mutations have been reported to be associated with the development of GBC (2, 11). Various groups have applied high-throughput proteomic approaches to study altered expression levels of proteins in tumor tissue from GBC patients. Tan et al. studied protein expression profiles of benign and GBC tissue using two-dimensional gel electrophoresis (2-DE) and identified 17 differentially expressed proteins (DEPs) (12, 13). The proteomic patterns of primary gallbladder cancer (PGC) in comparison to cholecystitis and normal gallbladder tissues using 2-DE revealed six DEPs (14). Another group applied iTRAQ-based quantitative proteomics using pooled GBC tissue lysate and identified 512 DEPs (15). However, the proteomic analysis using tumor tissue from early stage GBC is not yet performed.

In the present study, we have applied iTRAQ-based quantitative proteomic analysis to identify DEPs in early stage GBC in comparison to GSD (non-tumor controls) followed by verification of functionally relevant proteins by Western blot and IHC analysis.

## 2 Materials and methods

### 2.1 Clinical samples

Adult patients with age ≥ 20 years diagnosed with GBC or GSD cases (non-tumor control) visiting Govind Ballabh Pant Institute of Postgraduate Medical Education and Research (GIPMER), New Delhi, were recruited for the study. Clinical samples and data were also obtained from National Liver Disease Biobank- Institute of Liver and Biliary Sciences (NLDB-ILBS), New Delhi, India, after approval from the Maulana Azad Medical College- Institutional Ethics Committee, New Delhi (F.1/IEC/MAMC/80/08/2020/No. 314) and ICMR-National Institute of Pathology- Institutional Ethics Committee, New Delhi (NIP-IEC/10-12-19/06). All the participants provided written informed consent to participate in the study. Tumor Staging was done on the basis of clinical data of patients, histopathological evaluation and imaging tools, as per AJCC, 8^th^ edition staging system (7). Tissue samples from GBC cases (n=12) and GSD cases with no dysplasia (n=6) were used in this study. Tissue samples were collected immediately after surgical resection from patients with GBC or GSD and stored at -80° C until used for further analysis. Formalin fixed paraffin embedded (FFPE) tissue samples were used for ‘immunohistochemistry’ (IHC) analysis. Clinico-pathological data of these subjects are detailed in **Table 1**. Clinical parameters for the patients, wherever available (~50%), such as white cell count, liver enzymes (SGOT/SGPT/ALP) and cholestasis, and details of the sample used for quantitative proteomics and/or Western blot and/or IHC analysis are shown in **Supplementary data Table S1**.

**Table 1:** Clinico-pathological parameters of the patients used for the study.

Subjects	Total number	Number of males	Number of females	Mean age (Years)	Age range (years)
**Total GBC Cases**	24	5	19	51.5	27-65
**Stages**					
GBC, Stage I	8	3	5	51.5	38-65
GBC, Stage II	6	0	6	56.8	36-65
GBC, Stage III	6	2	4	42.7	27-65
GBC, Stage IV	4	0	4	56.8	47-61
**Histological grade**					
Well-differentiated (G1)	5	2	3	—	—
Moderately-differentiated (G2)	10	2	8	—	—
Poorly-differentiated (G3)	8	1	7	—	—
**LN status**					
LN negative	20	3	17	—	—
LN positive	4	2	2	—	—
**Controls- GSD**	16	2	14	46.6	24-68

### 2.2 Protein extraction

Tissue from individual cases (tumor tissue from GBC patients) or controls (GB tissue from GSD cases) was ground in liquid nitrogen followed by the addition of modified RIPA buffer with a 2% protease inhibitor cocktail. The tissue homogenate was then sonicated and centrifuged at 13,000 g for 20 min at 4°C. The supernatant was collected and protein estimation was done using the Bradford assay. SDS-PAGE was performed to analyze the protein profile of the tissue lysate from different groups and normalized the protein concentration based on total density.

### 2.3 iTRAQ labeling

For iTRAQ experiments, a pool of GSD tissue lysate (n=6) was used as a control while individual tissue lysate from GBC cases (n=7 for stage I and n=5 for stage II) was used for the analysis. For this, two iTRAQ experiments were performed. Experiment I included pooled GSD vs individual GBC cases (stage I) while Experiment II included pooled GSD vs individual GBC cases (stage II). The experimental design is shown in **Supplementary Figures S1, S2**.

For Experiment I, proteins (100 µg) from control (n=6, pooled sample) and GBC stage-I (n=7, individual samples) were reduced, alkylated and digested with trypsin followed by labeling of peptides with 8-plex iTRAQ reagents separately with specific iTRAQ labels (Reagent 113, 114, 115, 116, 117, 118, 119 and 121) as per the manufacturer’s instructions (iTRAQ Reagents Multiplex kit; Applied Biosystems). The labeled samples were pooled vacuum-dried and subjected to strong cation exchange (SCX) clean up (Cation exchange cartridge, Sciex, US), and desalted using a C18 column (Zorbax 300SB-C18, Agilent Technologies, US) as per the manufacturer’s instructions. The samples were then vacuum-dried and used for mass spectrometric analysis (nano-LC MS/MS analysis).

Similarly, for Experiment II, proteins (100 µg) from control (n=6, pooled samples) and GBC stage-II (n=5, individual samples) were reduced, alkylated and subjected to trypsin digestion and the peptides were labeled with 6-plex iTRAQ reagents separately with specific iTRAQ labels (Reagent 113, 114, 115, 116, 117 and 118) as mentioned above. The same pool of GSD samples was used as a control in both the iTRAQ experiments. The labeled samples were pooled vacuum-dried and subjected to SCX clean up and desalted using a C18 column followed by nano-LC MS/MS analysis.

### 2.4 LC-MS/MS analysis

Nanoflow electrospray ionization tandem mass spectrometric analysis was carried out using Orbitrap Fusion (Thermo Scientific, Bremen, Germany) interfaced with Easy-nLC 1000 nanoflow LC system. Peptides from each sample were enriched using a C18 trap column (75 μm × 2 cm) at a flow rate of 3 μl/min and fractionated on an analytical column (75 μm × 50 cm) at a flow rate of 280 nl/min using a linear gradient of 8-60% acetonitrile (ACN) over 46 min. Mass spectrometric analysis was performed in a data dependent manner with a cycle time of 3 seconds using the Orbitrap mass analyzer at a mass resolution of 120,000 at m/z 200. For each MS cycle, top most intense precursor ions were selected and subjected to MS/MS fragmentation and detected at a mass resolution of 50,000 at m/z 200. The fragmentation was carried out using higher-energy collision dissociation (HCD) mode. Normalized collision energy (CE) of 30% was used to obtain the release of reporter ions from all peptides detected in the full scan. The ions selected for fragmentation were excluded for the next 30 sec. The automatic gain control for full FT MS and FT MS/MS was set to 3e^6^ ions and 1e^5^ ions respectively with a maximum time of accumulation of 50 msec for MS and 75 msec for MS/MS. The lock mass with a 10 ppm error window option was enabled for accurate mass measurements (16). The LC-MS/MS analysis was performed three times for both experiments (I and II).

### 2.5 Identification and quantification of proteins

Protein identification, quantification and annotations of DEPs were carried out as described earlier by Priya et al. (16). The MS/MS data was analyzed using Proteome Discoverer (Thermo Fisher Scientific, version 2.2) with Mascot and Sequest HT search engine nodes using NCBI RefSeq database (release 89). Search parameters included trypsin as the enzyme with 2 missed cleavage allowed; precursor and fragment mass tolerance were set to 10 ppm and 0.1 Da, respectively; Methionine oxidation and deamidation of asparagines and glutamine amino acids was set as a dynamic modification while methylthio modification at cysteine and iTRAQ modification at N-terminus of the peptide and lysines were set as static modifications. The peptide and protein information was extracted using high peptide confidence and top one peptide rank filters. The FDR was calculated using percolator node in proteome discoverer 2.2. High confidence peptide identifications were obtained by setting a target FDR threshold of 1% at the peptide level. The labeling efficiency was > 95% for both the iTRAQ experiments (Stage I and II).

The iTRAQ intensity of proteins from each of the three replicates was used for the PCA plot analysis (17) to determine the correlation among the triplicate dataset as well as the correlation of GSD vs individual GBC stage I or stage II proteome dataset.

Relative quantitation of proteins was carried out based on the intensities of reporter ions released during MS/MS fragmentation of peptides. The proteins identified in all three replicates were used for the analysis. The average relative intensities of the two reporter ions for each of the unique peptide identifiers for a protein were used to determine the relative quantity of a protein and percentage variability. Proteins identified with ≥ 2 unique peptides, with 2-fold-change or above and FDR adjusted p value < 0.05 were considered significant and used for further analysis (16). The volcano maps were prepared by using log2 fold change and -log10 (p-value) as the co-ordinates and significant fold change ≥ 2.0 and p-value < 0.05 were considered to screen the proteins.

The data was analyzed for DEPs in individual patient with stage I or stage II and represented as Venn diagram. Further, the non-redundant list of DEPs in early stage GBC was derived and used for bioinformatics analysis.

### 2.6 Transcriptomics data comparison

We have compared the non-redundant list of DEPs from our study with the published transcriptome data in GBC (18–21). The proteins showing a positive correlation in their expression levels with transcriptome data are represented as scatter plot.

### 2.7 Bioinformatic analysis

Mapping of DEPs in early stage GBC (non-redundant list of DEPs from stage I and II) for localization, associated molecular functions, pathways and protein-protein interaction analysis was performed using the STRING (Search Tool for the Retrieval of Interacting Genes/Proteins) database (22). Signal sequence was predicted using SignalP software version 6.0 (https://services.healthtech.dtu.dk/service.php?SignalP) (23). From the non-redundant list of DEPs, the proteins with quantitation values for all 12 GBC patients were used for hierarchical clustering using Perseus software (17).

### 2.8 Western blot analysis

Western blot analysis was performed to further confirm the expression of myeloperoxidase precursor (MPO), myeloblastin precursor (PRTN3) and protein S100-A8 isoform d (S100A8) in the tissue lysates from individual GBC and GSD specimens (GBC stage I, n=7; GBC stage II, n=5; GSD, n=6). Briefly, tissue lysates were resolved on 12% SDS gel and transferred onto the PVDF membrane. Non-specific sites were blocked using 5% skimmed milk followed by incubation with primary antibody overnight (MPO, catalogue no. ab208670, dilution 1:4000; PRTN3, catalogue no. ab133613, dilution 1:10,000; S100A8, catalogue no. ab92331, dilution 1:2000). The blots were then incubated with secondary antibody (anti Rabbit-HRP, catalogue no. G-21234, 1/20,000) for 1 hr at RT and developed using the enhanced chemiluminescent (ECL) Kit (Millipore, USA) followed by image acquisition (24). The total density of the proteins in each lane was analyzed using densitometric analysis after SDS-PAGE analysis and was used for normalization (24). For quantitative analysis, the maximum density among GSD cases was considered to define the fold change in expression in individual GBC cases. The relative expression of target proteins in the individual GBC cases in Western blot analysis and quantitative proteomics data was represented as a bar diagram using Log2 fold change values.

### 2.9 Immunohistochemistry analysis

IHC was performed on FFPE tissues using individual tissue sections from controls (GSD cases), early stage GBC and advanced GBC cases (n=10 in each group) (**Supplementary Table S1**) to analyze the expression of MPO and S100A8 protein. IHC analysis was performed as described earlier by Akhtar et al. (25). In brief, after deparaffinization and rehydration of FFPE tissue sections, antigen retrieval was performed by immersing the slide in antigen retrieval buffer (20 mM Tris buffer, pH 9.0) at 90°C for 20 min. Endogenous peroxidases were blocked with 0.03% hydrogen peroxide, and nonspecific binding was blocked with protein blocking reagent. Sections were then incubated for 1 h at RT with primary antibody against MPO (dilution 1:8000, catalogue no. ab208670, Abcam, USA) and S100A8 (dilution 1:2000, catalogue no. ab92331) followed by incubation with PolyExcel PolyHRP for 40 minutes at RT. Tissue sections were then incubated with Stunn DAB working solution for 5 min at RT (PathnSitu Biotechnologies, USA). Sections were counter stained with Mayer’s hematoxylin, dehydrated and images were taken under the microscope. The distribution of staining and staining intensity across the section was observed under the microscope. For MPO, the number of neutrophils was counted and ≥20 was considered as ‘Positive’, while <20 was considered as ‘Negative’. For S100A8, scoring criteria were based on both staining intensity and distribution. The 2+ or higher intensity, with ≥10% distribution was considered as ‘Positive’, while 1+ positivity or < 10% distribution was considered as ‘Negative’. IHC data analysis was done by two independent pathologists.

The statistical analysis (Fisher’s exact test) was performed using GraphPad Prism 5 (26) to study the correlation of MPO and S100A8 expression among cases and controls (early stage GBC vs controls; advanced stage vs controls; all GBC vs controls). The *p*-value less than 0.05 indicated statistical significance.

## 3 Results

**Figure 1:**
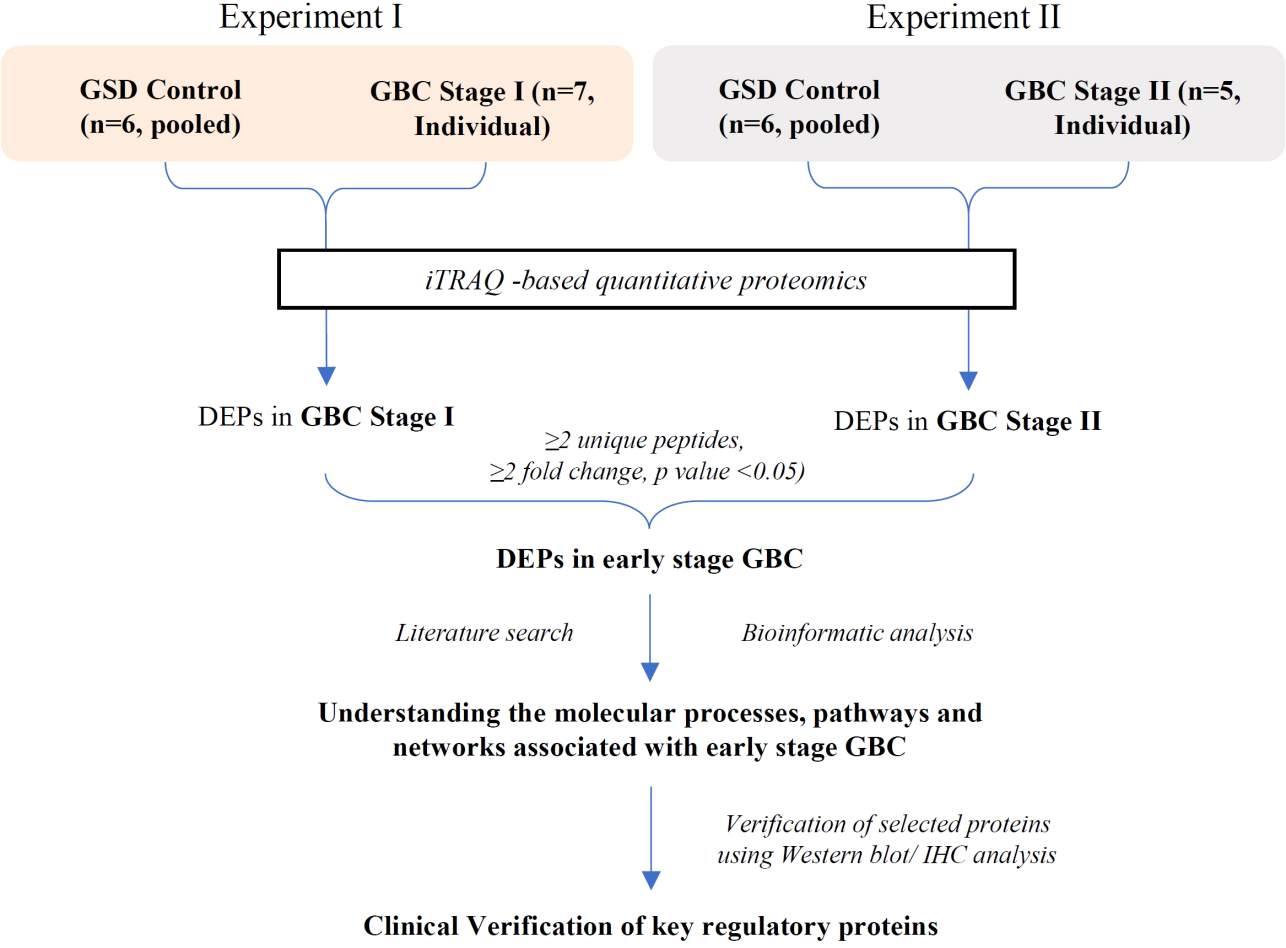
Overall workflow of the study. GSD, Gallstone disease; GBC, Gallbladder cancer; DEPs, Differentially expressed proteins.

In the present study, we performed the differential protein profiling of tumor tissue from early stage GBC cases to identify the proteins and associated molecular pathways. The overall work plan of the study is shown in **Figure 1**.

### 3.1 Identification of differentially expressed proteins in early stage GBC

**Figure 2:**
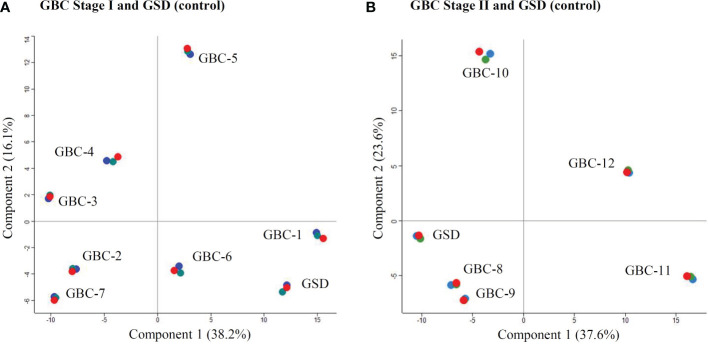
PCA Plot showing the correlation of the individual patients with GBC stage I and II. **(A)** includes seven individual samples from GBC stage I and one pooled GSD control while **(B)** includes five individual samples from GBC stage II along with one pooled GSD control. Four patients, two from stage I (GBC-1 and 6) **(A)** and two from stage II (GBC-8 and 9) **(B)** showed similar profile as GSD (non-tumor control). The technical replicates showed a significant correlation. Replicates R1, R2 and R3 are shown in red, green and blue color. The PCA plot is derived using the iTRAQ reporter intensity from the quantitative proteomics data.

We performed iTRAQ based LC-MS/MS analysis of 12 early stage GBC patients (stage I-n=7, stage II- n=5) using two independent experimental setups. The experimental setup-1 consists of 7 GBC - stage I samples vs pooled GSD samples while the experimental setup-2 consists of 5 GBC -stage II samples vs pooled GSD samples (**Figure 1**, **Supplementary Figure S1, S2**). The analysis led to the identification of a total of 1450 proteins from stage I and 2662 proteins in stage II. PCA plot analysis of the proteome profile of 12 GBC patients along with GSD control showed a significant correlation among the three replicate datasets of each stage (**Figure 2**). We found 184 DEPs with ≥ 2 fold change and adjusted p-value ≤0.05 in GBC stage I (**Supplementary Table S2**) while a total of 256 DEPs with ≥ 2 fold change and adjusted p-value ≤0.05 were identified in GBC stage II (**Supplementary Table S3**).

We analyzed the DEPs across individual patients and the data is represented as Volcano plots in **Supplementary Figure S3**. The analysis showed a total of 357 DEPs (non-redundant) in early stage GBC (stage I and II). We further compared our proteomics data with the published transcriptome data in GBC and found 97 proteins mapping with the transcriptome data. Of these, 71 proteins (73%) showed a positive correlation with transcript data. The proteins showing positive correlation are represented in **Supplementary Figure S4**.

**Figure 3:**
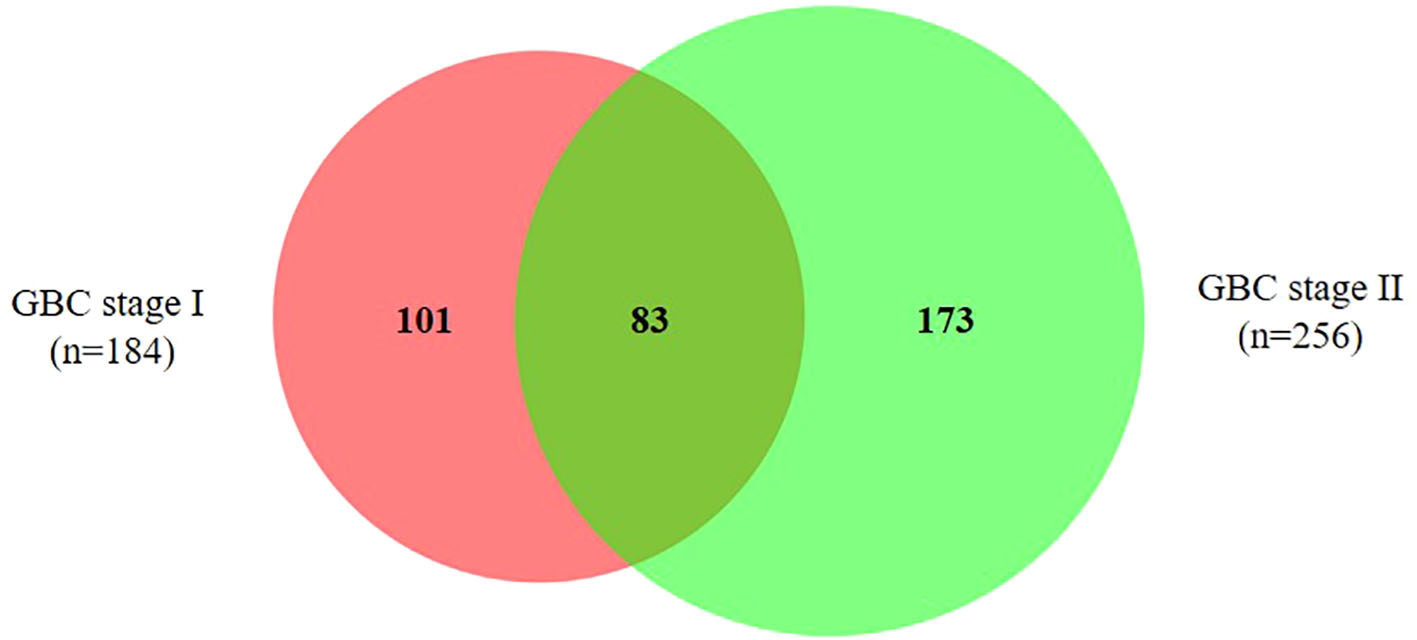
Venn diagram showing DEPs in early stage GBC. A total of 83 proteins are common to both stage I and II, while 101 proteins are specific to stage I and 173 proteins are specific to stage II. The details of all the proteins are shown in **Supplementary Table S4**.

Out of 357 DEPs, a total of 83 proteins are common to both stage I and II, while 101 proteins are specific to stage I and 173 proteins are specific to stage II (**Figure 3**, **Supplementary Table S4**). Out of 83 DEPs, the majority of the proteins (~95%) showed a similar trend (up or down) of expression in both stages. A total of 29 proteins were found to be differentially expressed in ≥ 50% GBC cases (i.e. 6 patients) and are shown in **Table 2**. Some of the functionally relevant proteins include Myeloperoxidase precursor (MPO), Myeloblastin precursor (PRTN3), Neutrophil defensin 1 isoform X1 (DEFA1), Protein S100-A8 isoform d (S100A8), Desmin (DES), creatine kinase B-type isoform 2 (CKB), Transgelin (TAGLN), Annexin A3 (ANXA3).

**Table 2:** A list of 29 DEPs in ≥ 6 early stage GBC patients.

	GBC Stage I							GBC Stage II							
Gene Symbol	GBC-1	GBC-2	GBC-3	GBC-4	GBC-5	GBC-6	GBC-7	GBC-8	GBC-9	GBC-10	GBC-11	GBC-12	No. of GBC stage I patients with DE	No. of GBC stage II patients with DE	No. of early stage GBC patients with DE
ALB	1.25	**0.41**	**0.21**	**0.29**	0.87	**0.44**	**0.40**	**0.46**	**0.40**	0.65	**0.18**	0.53	5	3	8
ANXA3	1.44	**2.60**	**3.76**	**3.91**	2.43	1.23	**2.45**	1.19	1.04	**2.71**	**2.30**	**2.51**	4	3	7
AOC3	0.69	**0.24**	**0.29**	**0.21**	**0.26**	**0.42**	**0.18**	0.72	1.56	0.35	**0.29**	**0.28**	5	2	7
BGN	1.21	**0.40**	**0.39**	**0.47**	**0.29**	0.72	**0.38**	1.06	0.93	0.42	**0.44**	**0.34**	5	2	7
CKB	**0.41**	**0.46**	**0.35**	**0.42**	**0.26**	**0.29**	**0.39**	**0.47**	0.88	0.51	**0.42**	**0.29**	7	3	10
COL6A1	1.29	0.41	0.37	**0.38**	**0.27**	**0.43**	**0.38**	**2.64**	1.12	0.54	0.72	**0.41**	4	2	6
DCN	**2.06**	**0.29**	**0.26**	**0.29**	**0.27**	0.73	**0.29**	0.62	1.42	0.48	**0.30**	**0.30**	6	2	8
DEFA1	2.61	**3.61**	**10.42**	**2.59**	4.62	2.16	4.01	**3.62**	0.94	**4.95**	**4.54**	**3.12**	3	4	7
DES	0.40	**0.09**	**0.09**	**0.08**	**0.07**	**0.09**	**0.08**	**0.48**	**0.27**	0.12	**0.10**	**0.09**	6	4	10
FLNA	0.66	**0.47**	**0.40**	**0.39**	**0.35**	0.71	**0.47**	0.93	0.56	0.33	**0.34**	**0.37**	5	2	7
HBB	0.70	**0.31**	**0.26**	1.30	0.79	**0.42**	**0.48**	0.57	**0.50**	**7.28**	**0.50**	0.52	4	3	7
HSP90B1	0.64	**2.60**	2.22	1.86	1.09	**3.24**	**2.40**	1.68	**2.15**	1.07	**2.06**	**2.11**	3	3	6
HSPA5	0.97	**2.52**	**2.26**	**2.13**	1.16	**2.46**	**2.19**	1.49	1.71	1.11	**2.03**	1.73	5	1	6
HSPE1	0.80	**3.02**	**3.50**	**3.35**	1.42	1.51	**2.54**	0.98	1.26	1.04	**4.45**	**3.37**	4	2	6
KRT18	**0.48**	1.18	**3.18**	**2.65**	1.41	0.48	**2.43**	**0.45**	**0.43**	0.33	0.69	1.58	4	2	6
KRT8	0.61	**2.24**	**3.61**	**2.09**	1.30	**0.42**	1.93	**0.44**	**0.41**	0.40	0.96	1.87	4	2	6
LUM	1.89	**0.17**	**0.15**	**0.31**	**0.20**	0.84	**0.17**	0.55	1.52	0.42	**0.20**	**0.23**	5	2	7
MPO	2.53	**2.57**	**6.15**	**2.60**	**10.27**	1.60	**2.97**	**2.78**	1.28	2.82	**3.09**	1.16	5	2	7
MYL6	0.80	**0.30**	**0.27**	**0.49**	**0.37**	0.58	**0.28**	0.67	1.03	0.25	**0.46**	**0.38**	5	2	7
MYL9	0.61	**0.18**	**0.14**	**0.23**	**0.12**	**0.39**	**0.14**	0.57	0.98	0.16	**0.18**	**0.15**	6	2	8
P4HB	0.99	**2.53**	**3.83**	**2.43**	1.53	**2.94**	**3.53**	1.23	1.23	0.95	1.31	**2.93**	5	1	6
PRELP	1.45	**0.21**	**0.19**	**0.22**	0.27	**0.45**	0.20	1.16	1.56	0.47	**0.25**	**0.31**	5	2	7
PRTN3	3.59	**11.49**	**30.01**	**13.09**	16.06	2.94	**7.52**	**2.82**	1.45	**16.93**	**2.40**	**2.23**	4	4	8
S100A8	3.43	**3.51**	**4.97**	2.12	**6.96**	**2.49**	**4.66**	**2.80**	0.81	1.99	**2.25**	1.42	5	2	7
SERPINA1	0.37	**0.25**	**0.28**	**0.41**	0.89	1.12	**0.43**	**0.29**	0.62	0.75	**0.26**	0.66	4	2	6
SOD2	1.03	**2.75**	**2.91**	**11.37**	1.53	**5.06**	**2.31**	1.60	**2.05**	1.07	1.25	**2.99**	5	2	7
TAGLN	0.75	**0.17**	**0.14**	**0.15**	**0.13**	0.54	**0.20**	0.80	0.74	**0.29**	**0.15**	**0.19**	5	3	8
TPM2	**0.41**	**0.12**	**0.08**	**0.11**	**0.09**	**0.27**	**0.12**	0.56	1.13	0.22	**0.19**	**0.20**	7	2	9
TPSAB1	0.82	**0.28**	**0.20**	**0.21**	**0.17**	**0.47**	**0.22**	0.77	0.98	**0.30**	**0.19**	**0.23**	6	3	9

The proteins marked in bold are DE with 2 fold change, adjusted p value < 0.05 and identified with ≥ 2 unique peptides. ND- Not detected, DEPs- Differentially expressed proteins.

### 3.2 Signal sequence analysis and literature survey

The Signal sequence analysis of 357 proteins showed 109 proteins with a signal sequence. Literature survey showed a total of 106 proteins that are reported to be differentially abundant in plasma or serum in cancer. Overall, we found 51 proteins to have signal sequence as well as reported to be differentially abundant in plasma or serum in cancer (**Supplementary Figure S5**, **Supplementary Table S5**). These proteins are potential circulatory markers for the detection of GBC

### 3.3 Bioinformatic analysis

**Figure 4:**
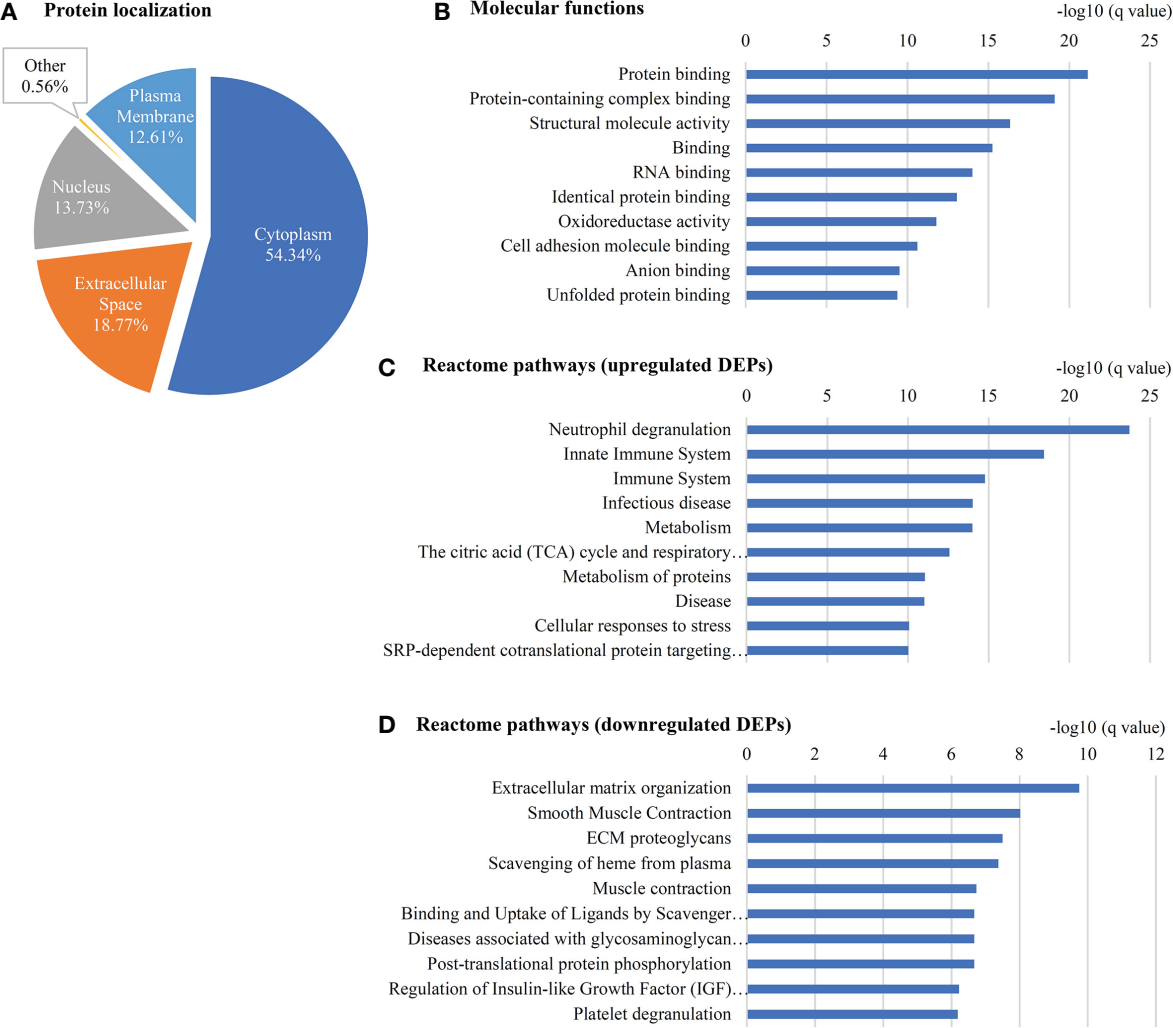
Gene ontology of 357 DEPs in early stage GBC. **(A)** Localization of **(B)** Molecular functions **(C)** Reactome pathways using upregulated proteins and **(D)** downregulated proteins as observed using STRING database.

A gene ontology analysis for the localization of 357 DEPs showed that 54.3% of them belong to the cytoplasm, 18.8% are from the extracellular region, 13.7% are associated to the nucleus, 12.6% are from the plasma membrane and less than 1% are associated with other localization (**Figure 4A**). The top molecular functions include Opsonin binding, MHC class II protein complex binding, Lipase inhibitor activity, Lipoprotein particle receptor binding and MHC class I protein binding (**Figure 4B**, **Supplementary Table S6**). Pathway analysis using 191 upregulated proteins showed ‘Neutrophil degranulation’ among the top upregulated pathway (**Figure 4C**, **Supplementary Table 7A**) while the analysis using 62 downregulated proteins showed ‘ECM organization’ to be the top downregulated pathway (**Figure 4D**, **Supplementary Table 7B**).

**Figure 5:**
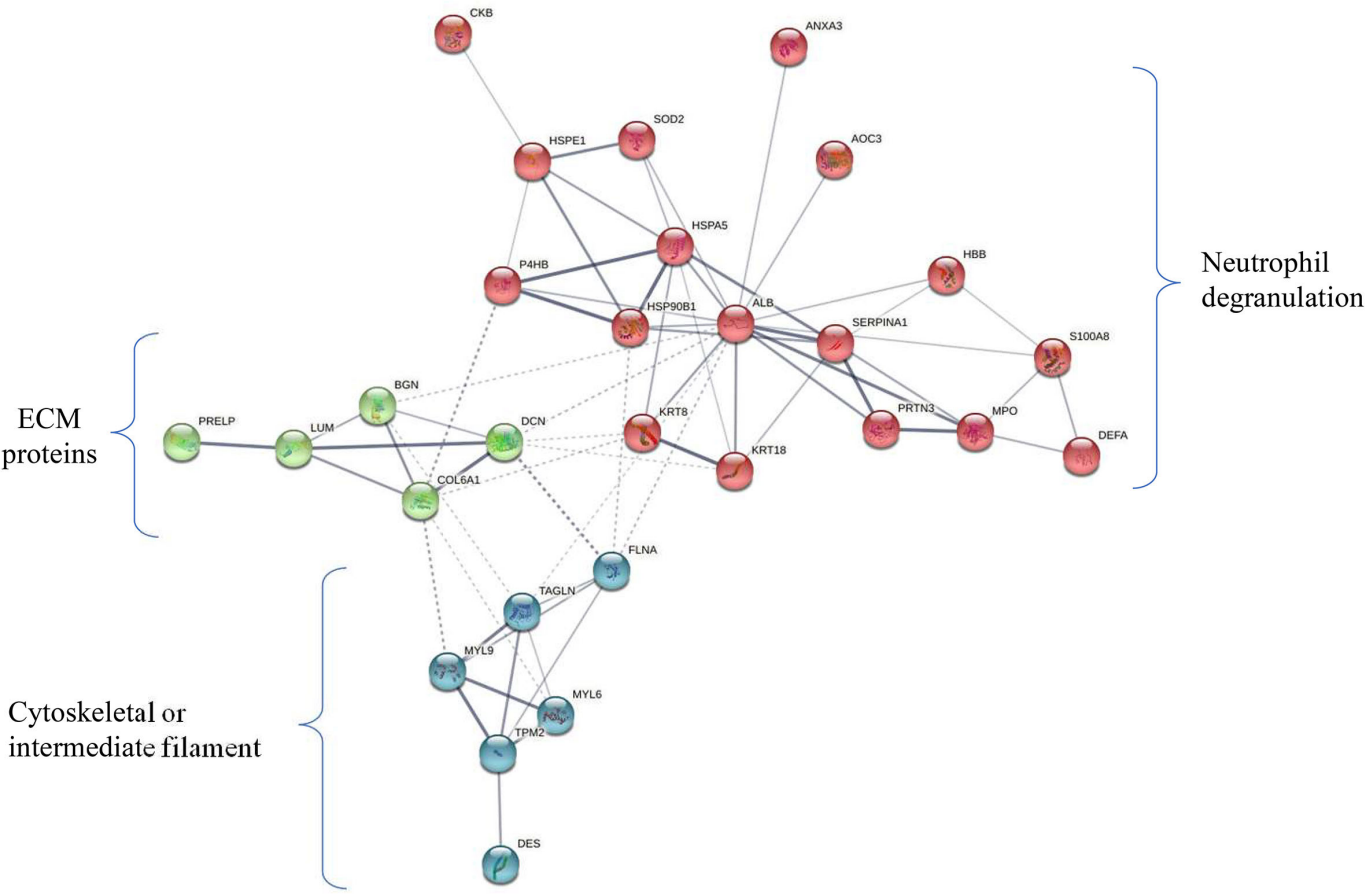
Protein-protein-interaction (PPI) network of 29 deregulated proteins. PPI analysis showed four clusters including majorly the proteins associated with neutrophil degranulation (MPO, DEFA1, S100A8, PRTN3, AOC3) (marked in red), ECM proteins (COL6A1, BGN, DCN, LUM, PRELP) (green), cytoskeletal or intermediate filament (DES, MYL6, MYL9, TPM2) (blue). The subset of 29 proteins showed differential expression in ≥ 50% of early stage GBC (i.e. ≥ 6 patients).

Protein-protein interaction analysis of 29 proteins (DE in ≥ 6 patients) revealed three clusters which include the proteins associated with neutrophil degranulation (MPO, DEFA1, S100A8, PRTN3, AOC3), ECM proteins (COL6A1, BGN, DCN, LUM, PRELP) and cytoskeletal or intermediate filament (DES, MYL6, MYL9, TPM2) (**Figure 5**).

We also performed the pathway analysis using the DEPs across individual patients and obtained the data for 10 out of 12 patients. ‘Neutrophil degranulation’ was among the top pathways in 9 patients (**Supplementary Table S8**). The data for two patients was not obtained as the number of DEPs was low.

**Figure 6:**
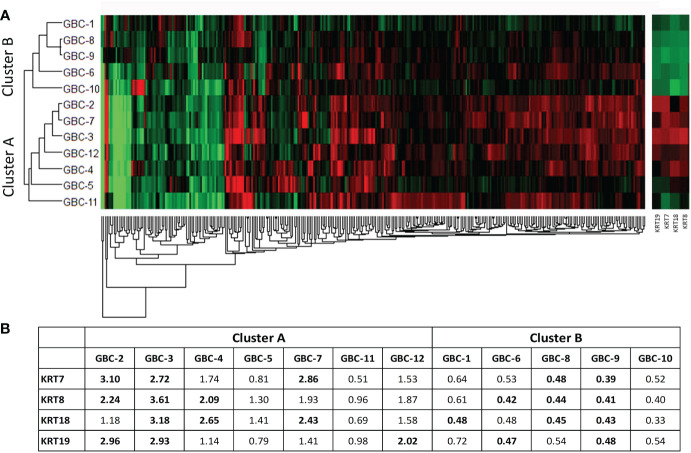
Hierarchical clustering using non-redundant list of 308 DE proteins in 12 early stage GBC patients. **(A)** Hierarchical clustering showed two clusters with cluster A majorly including stage I samples and cluster B majorly including stage II samples. **(B)** We observed cytokeratins KRT7, KRT8, KRT18 and KRT19 showing upregulation in Cluster A and downregulation in Cluster B. Log2 (fold change) values for 308 proteins were used for the analysis. Red- Upregulated, Green- Downregulated.

A hierarchical clustering analysis done using a non-redundant list of 308 proteins with quantitation value for all the 12 patients showed two distinct clusters or groups on the basis of their molecular profile (**Figure 6A**). The majority of Stage I and II samples were clustered and represented as Cluster A and B respectively. Among 308 proteins, we observed keratin family proteins (KRT7, KRT8, KRT18 and KRT19) to be upregulated in cluster A and downregulated in cluster B (**Figure 6B**).

### 3.4 Validation of target protein expression by western blot and immunohistochemistry analysis

**Figure 7:**
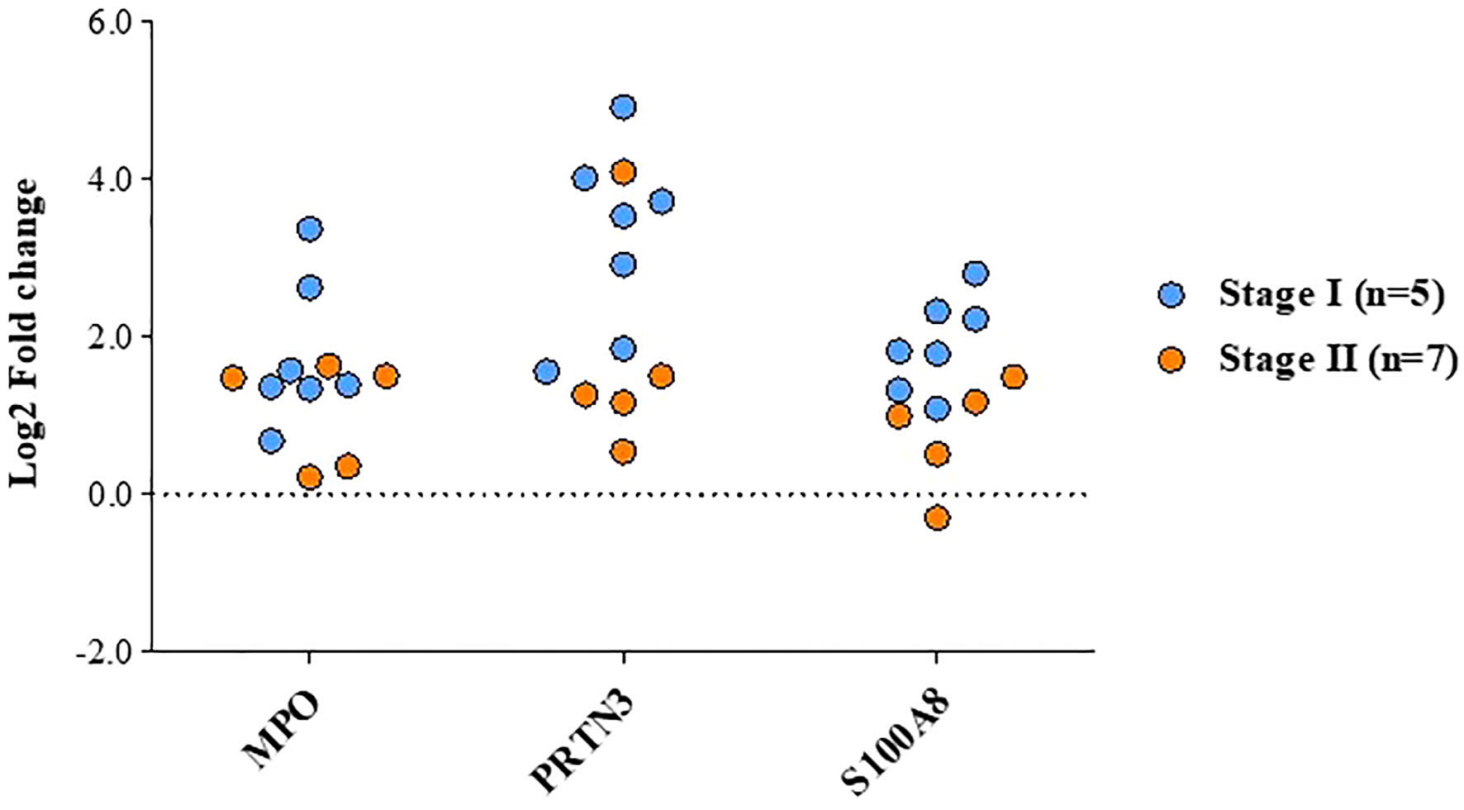
Altered levels of functionally relevant proteins in early stage GBC as observed in quantitative proteomics data. The plot showing the levels of MPO, PRTN3 and S100A8 in individual patients, GBC stage-I (n=7) and stage-II (n=5).

**Figure 8:**
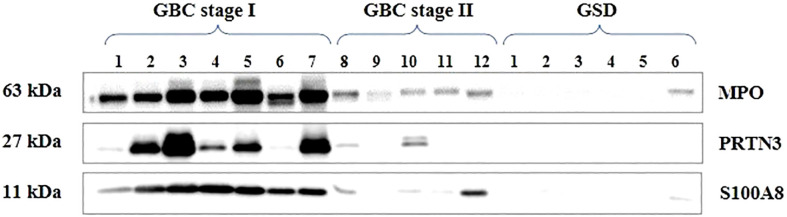
Western blot images showing expression of MPO, PRTN3, S100A8 in the individual tissue samples from early stage GBC and GSD cases. A significant overexpression of MPO, PRTN3, S100A8 was found in 66.7% (n=8/12), 66.7% (n=8/12) and 83.3% (10/12) early stage GBC cases respectively.

We selected three proteins (MPO3, PRTN3 and S100A8) based on their association with ‘neutrophil degranulation pathway’ and ‘overexpression in ≥6 patients in quantitative proteomics data’ for validation by Western blot analysis. Their relative expression (log2 fold change) in individual patients from the quantitative proteomics dataset is shown in **Figure 7**. Western blot analysis was performed using individual tissue lysates and showed overexpression of MPO, PRTN3 and S100A8 in early stage GBC cases and GSD controls. Both MPO and PRTN3 showed significant overexpression in 66.7% (n=8/12) of the GBC cases whereas there was a weak or no signal observed in GSD. The protein S100A8 showed significant overexpression in 83.3% (n=10/12) of the GBC cases in comparison to GSD. Western blot image is shown in **Figure 8** and full-length blot image is shown in **Supplementary Figure S6**. The relative expression of selected proteins in the individual GBC cases using Western blot analysis and quantitative proteomics data is shown in **Supplementary Figure S7**.

**Figure 9:**
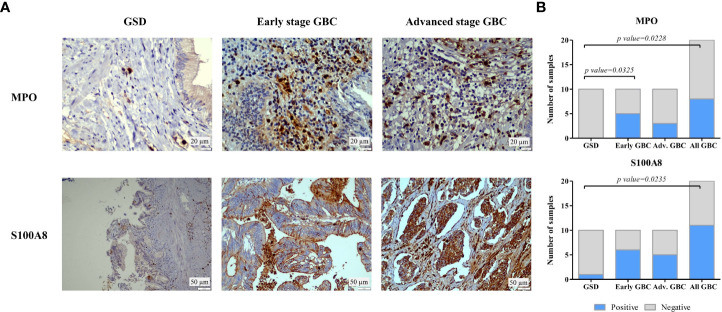
IHC analysis to study the expression of MPO and S100A8 in controls and GBC cases. **(A)** Representative IHC images showing the expression of MPO and S100A8 in controls and GBC cases. IHC was performed on formalin-fixed paraffin-embedded (FFPE) individual tissue sections of 10 controls (GSD cases with no dysplasia), 10 early stage GBC (stage I and II) cases and 10 advanced stage GBC cases (stage III and IV). The IHC results showed that the number of MPO positive neutrophils was found to be ‘positive’ in 50% of early stage GBC and 30% of advanced stage GBC cases. All GSD cases showed ‘negative’ expression. The expression of S100A8 was found to be ‘positive’ in 10% GSD cases, 60% early and 50% advanced stage GBC. **(B)** The statistical analysis between cases and controls showed a significant difference of MPO positive neutrophils in early stage GBC vs controls and all GBC vs controls while a significant difference of S100A8 was observed in all GBC vs controls. The controls (≥ 90%) showed ‘Negative’ expression levels.

We performed IHC analysis to study the expression of two of the proteins, MPO and S100A8 in controls, early stage GBC and advanced stage GBC (n=10 in each group). **Figure 9A** shows the representative IHC images of controls, early stage GBC and advanced stage GBC. The number of MPO positive neutrophils was found to be ‘positive’ in 50% of early stage GBC and 30% of advanced stage GBC cases. All GSD cases showed ‘negative’ expression. The expression of S100A8 was found to be ‘positive’ in 10% GSD cases, 60% early and 50% advanced stage GBC. The statistical analysis between cases and controls showed a significant difference (p value ≤0.05) of MPO positive neutrophils in early stage GBC vs controls and all GBC vs controls while a significant difference of S100A8 was observed in all GBC vs controls (**Figure 9B**). The controls (≥ 90%) showed ‘Negative’ expression levels. We performed IHC analysis for PRTN3, however, the results were not clear due to technical reasons.

## 4 Discussion

GBC is generally diagnosed at advanced stages and has a poor prognosis. The detection of the disease at the early stage may significantly improve the treatment strategy and survival outcome of the patients. There are few studies applying high throughput proteomics approach to understand the molecular processes in GBC (12–15), however, none of these focused on early stage GBC. The present study applied iTRAQ-based quantitative proteomics approach and analyzed the differential proteome in early stage GBC (stage I and II). The data from both the stages were combined to obtain a non-redundant list of DEPs. The correlation of expression between these DE proteins (our study) and DE transcript dataset in GBC available in the public domain was analyzed. Further, gene ontology analysis was carried out to identify the significantly altered pathways. Based on the pathway analysis, we propose a hypothesis on the dysregulated molecular processes/events in early stage GBC. We then analyzed the proteins for the presence of ‘signal sequence’ to identify those having the potential for early detection of GBC.

The present study identified a non-redundant list of 357 DEPs in early stage GBC, of these, 68 proteins are reported earlier in GBC including KRTs (KRT7, KRT8, KRT18 KRT19, KRT20), VIM, DES, CEACAM5 or CEA, S100A8, TAGLN, HMGB1, ANXA3, while others are novel to GBC. A total of 272 proteins are reported to be differentially expressed in other cancers and 17 are novel. Comparison with the already published transcriptome dataset showed 97 proteins mapping with the transcriptome data, of which 71 proteins (73%) showed a positive correlation in expression. Pathway analysis showed ‘neutrophil degranulation’ to be the top upregulated pathway and ‘ECM organization’ to be the top downregulated pathway in early stage GBC. The individual patient data analysis showed 29 DEPs in ≥ 50% of GBC cases (≥ 6 patients) (**Table 2**). Some of the proteins associated with neutrophil degranulation such as MPO, MMP9, DEFA1 showed the presence of ‘signal sequence’ and could be the potential circulatory markers for early detection of GBC.

Immune cell infiltration (neutrophils, macrophages) is well reported in several cancers. Neutrophils are associated with cancer-related inflammation with a dual role in pro and anti-tumor effects (27). In different types of cancer, neutrophils have been reported to have pro-tumorigenic properties *via* DNA damage, immunosuppression and angiogenesis, which contribute to the progression of the disease in the tumor microenvironment (TME) (28). We found overexpression of PRTN3 (proteomics data) which is reported to be associated with neutrophil trans-endothelial migration to the tissue (29). We observed an overexpression of neutrophil intracellular marker protein (MPO) and cell surface marker proteins (CEACAM8, ITGAM and ITGB2) in early stage GBC in comparison to GSD (non-tumor controls) suggesting neutrophil infiltration in tumor tissue. Increased expression of CEACAM8, ITGAM and ITGB2 is reported to be associated with exocytosis or degranulation of primary, secondary and tertiary neutrophil granules respectively. We also found overexpression of various neutrophil granule proteins including primary granule (Azurophil) proteins such as AZU1, DEFA1, PRTN3, CD63, CTSG, ELANE, MPO, secondary granule proteins such as LCN2, LTF, tertiary granules (Gelatinase) such as MMP9 and other granule proteins such as S100A8 and S100A9, in the early stage GBC (our proteomics data). As per the HPA data, AZU1, DEFA1, PRTN3, CTSG, ELANE, MPO, LTF, MMP9, S100A8 and S100A9 are bone marrow and lymphoid tissue specific or enriched proteins suggesting that the expression of these proteins detected in our data is from immune cells.

MPO is a member of the heme peroxidase superfamily and is the most abundant protein expressed by neutrophils. It is reported to generate reactive oxygen species (ROS) leading to DNA damage and mutation inducing carcinogenesis and thus resulting in tissue damage (30). PRTN3 is a serine protease secreted by cells of myeloid lineage and allocated to the cell surface of neutrophils and endothelial cells. It has an elastase-like specificity for small aliphatic residues such as Ala, Val, Ser, Met and degrades various ECM proteins and known to activate MMP and is associated with tumor invasion and metastasis (31). S100A8 is a calcium-binding S100 protein secreted by granulocytes and monocytes. S100A8 has emerged as an inflammatory factor and is associated with cancer. S100A8 overexpression is associated with tumorigenesis and poor differentiation in melanoma and prostate cancers, although the biological function of S100A8 in cancer is not clear (32). Western blot analysis confirmed the overexpression of tissue MPO, PRTN3 and S100A8 in early stage GBC cases (**Figure 8**) and IHC analysis confirmed the overexpression of MPO in early stage GBC.

**Figure 10:**
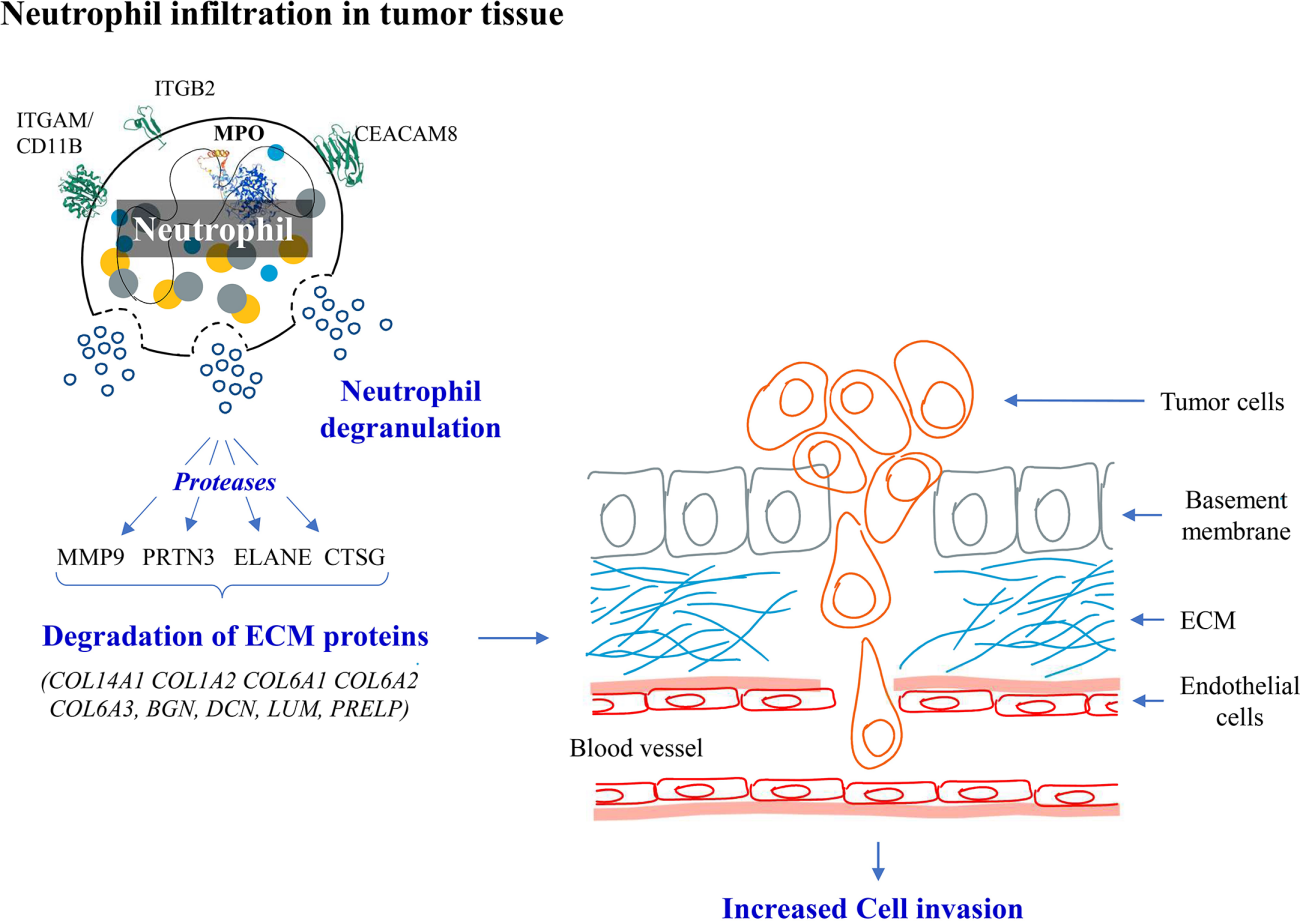
Hypothesis showing molecular events in early stage GBC. We observed overexpression of neutrophil degranulation pathway proteins and downregulation of ECM proteins. We hypothesize that there is neutrophil infiltration and degranulation in GBC tissue resulting in release of proteases which possibly degrades ECM proteins promoting cancer cell invasion.

PRTN3, ELANE, CTSG and MMP9 are the serine proteases released by the activated neutrophils and have been reported to degrade ECM proteins and promote cancer cell invasion (33–36). We also observed downregulation of ECM proteins (COL14A1, COL1A2, COL6A1, COL6A2, COL6A3, BGN, DCN, LUM, PRELP). Majority of these proteins are already reported to be involved in cell invasion. Based on the above results, we hypothesize that there is an increased neutrophil infiltration and degranulation in the tumor tissue leading to degradation of ECM proteins and promoting cancer cell invasion in early stage GBC (**Figure 10**). Protein-protein interaction analysis of the proteins associated with ‘neutrophil degranulation’ showed MPO, ELANE, ITGAM, MMP9, LTF to be the hub molecules (**Supplementary Figure S8**). Based on the bioinformatic analysis and literature search, some of the proteins associated with neutrophil degranulation such as MPO, ELANE, DEFA1, MMP9 were found to have ‘signal sequence’ and could be further explored as circulatory markers for early detection of GBC.

The limitations of the study include the low sample size. We used GSD cases as non-tumor controls in the present study, however, inclusion of other controls such as GB polyp, xanthogranulomatous cholecystitis would be important.

## 5 Conclusions

In the present study, we analyzed the differential proteome profile of early stage GBC patients and identified 357 differentially expressed proteins. ‘Neutrophil degranulation’ pathway was found to be enriched with upregulated proteins and ‘ECM organization’ with downregulated proteins. We hypothesize that there is neutrophil infiltration and degranulation in tumor tissue which leads to degradation of ECM proteins and promote tumor progression from early stage GBC. The overexpression of ‘neutrophil degranulation’ pathway proteins was further confirmed by Western blot and IHC analysis. The neutrophil degranulation proteins having signal sequences identified in the present study could be explored as circulatory markers for early detection of GBC.

## Data availability statement

The original contributions presented in the study are included in the article/**Supplementary Material**. Further inquiries can be directed to the corresponding authors.

## Ethics statement

The studies involving human participants were reviewed and approved by Maulana Azad Medical College- Institutional Ethics Committee, New Delhi and ICMR-National Institute of Pathology- Institutional Ethics Committee, New Delhi. The patients/participants provided their written informed consent to participate in this study.

## Author contributions

PG, PS designed the experiment. PS, AA, SG, JA, VJ and RP contributed to clinical sample collection and clinical data management. JA and VJ did experimental work and data compilation. Mass spectrometric data acquisition and analysis: VG, JA, VJ. Literature search and interpretation of overall data was done by PG, RS, RP, PS, AA, SG, SK, JA, VJ, RP, RK. Drafting and editing of the manuscript was done by PG, JA, PS, AA, RS, RP, SK, VG. All authors read and approved the final manuscript. All authors contributed to the article and approved the submitted version.
