# Supplementary material for: Quantitative tissue proteome profile reveals neutrophil degranulation and remodeling of extracellular matrix proteins in early stage gallbladder cancer
Source: Front Oncol. 2023 Jan 6;12:1046974. doi: 10.3389/fonc.2022.1046974 (PMC9853450; doi:10.3389/fonc.2022.1046974)
Supplement: Supplementary file 1 [file DataSheet_1.pdf]

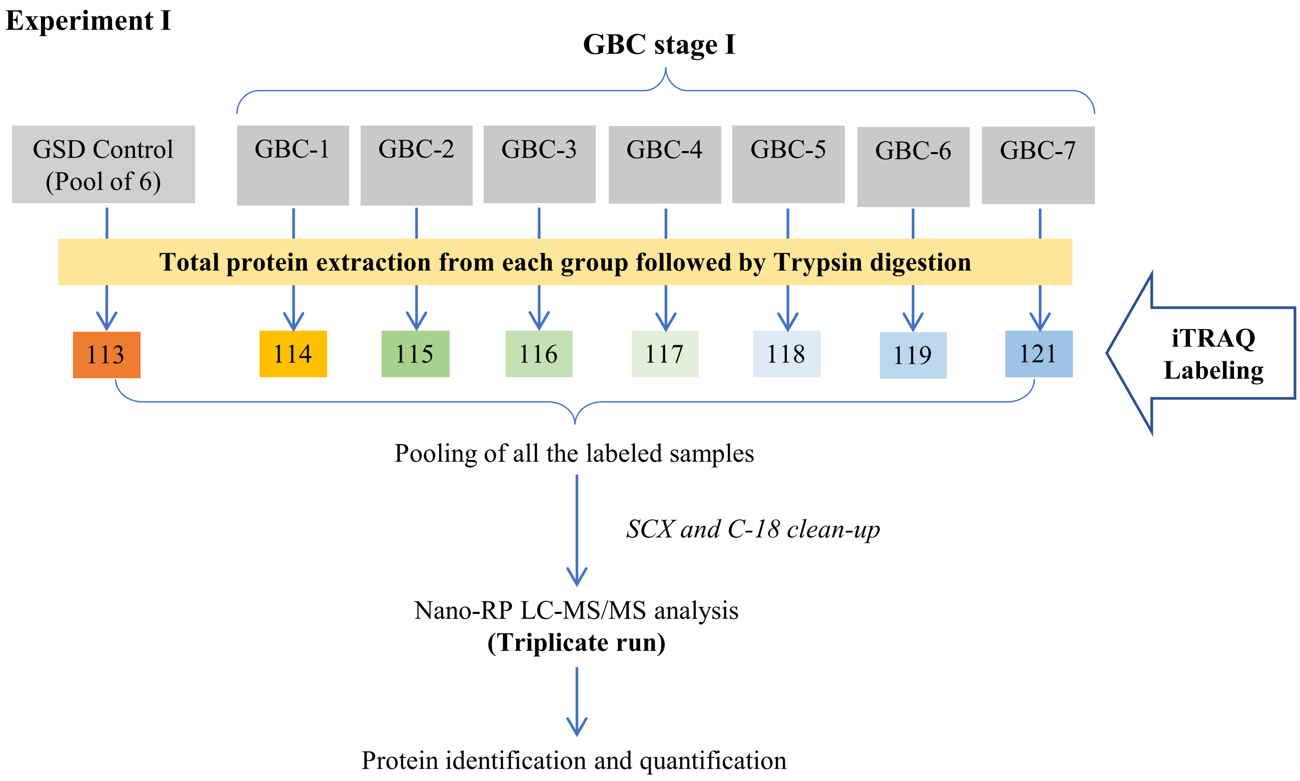

Supplementary Figure S1

Experimental design of iTRAQ-based quantitative proteomic analysis for identification of DEPs in GBC stage I

Experiment II

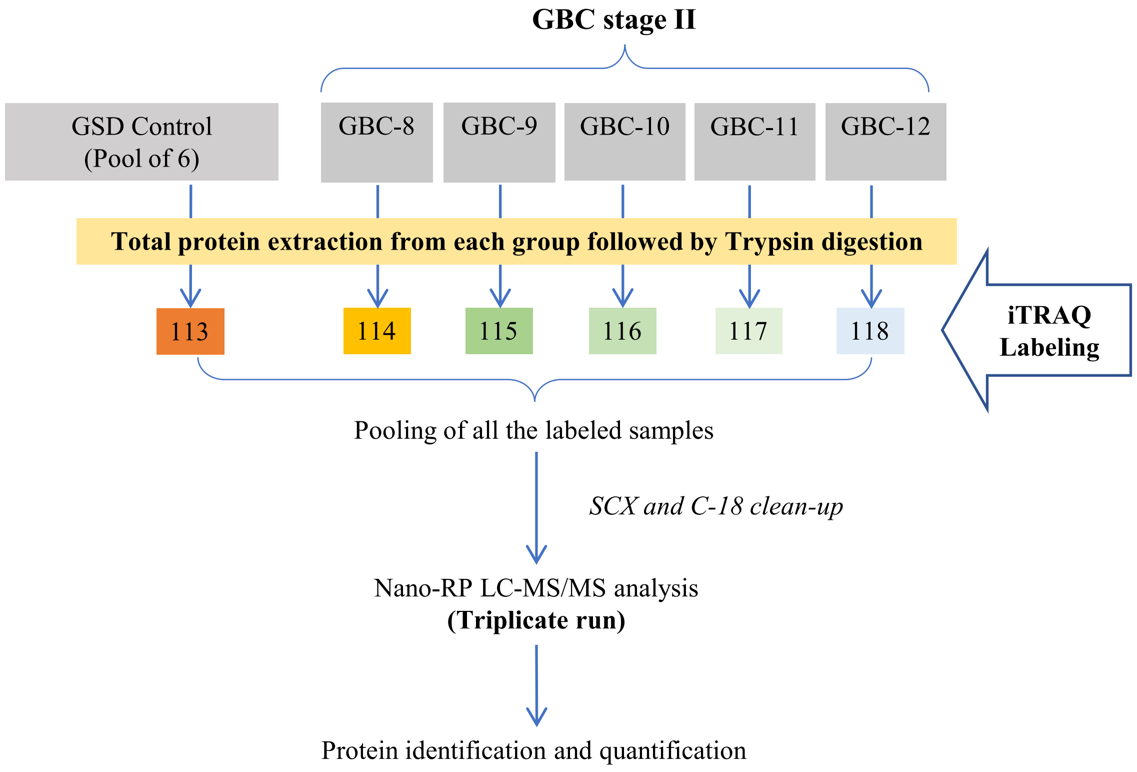

Supplementary Figure S2

Experimental design of iTRAQ-based quantitative proteomic analysis for identification of DEPs in GBC stage II

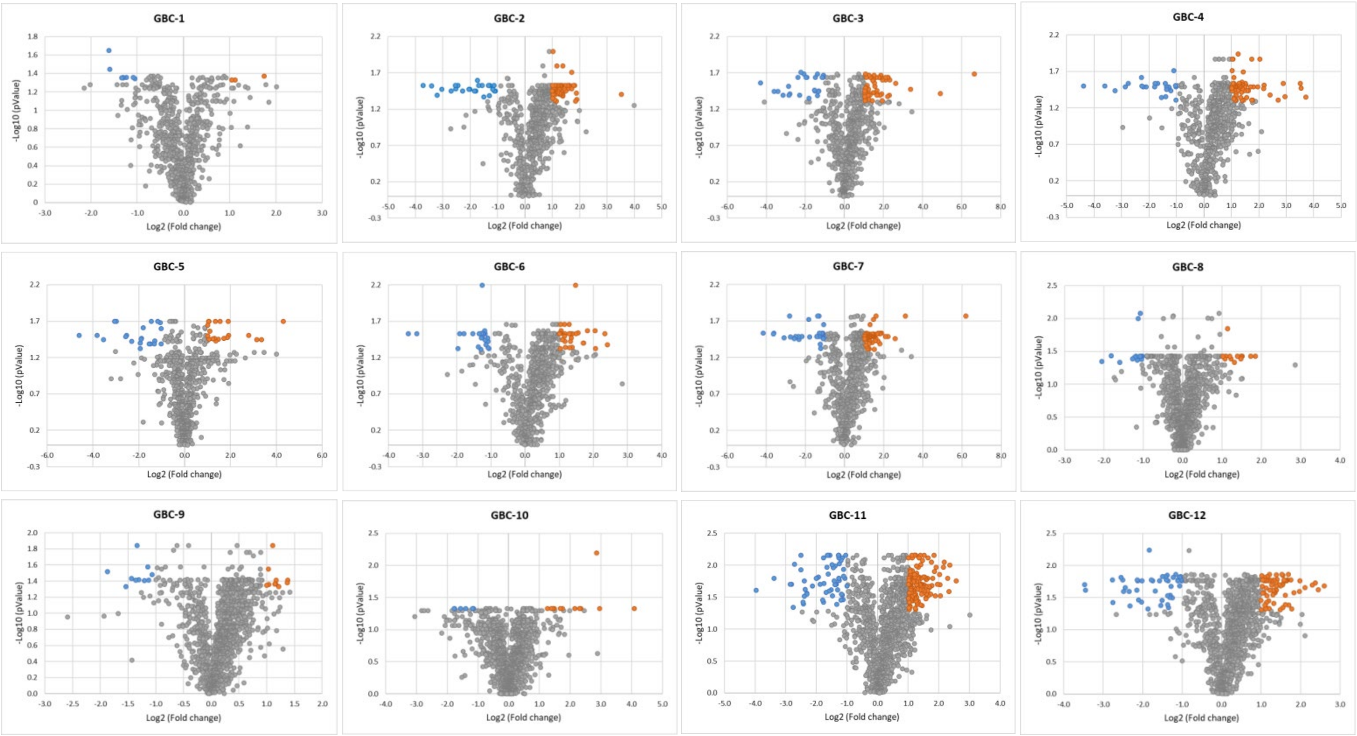

Supplementary Figure S3

Volcano plot showing DEPS in individual GBC patients. The volcano map was prepared by using log2 fold change and -log10 (p-value) as the co-ordinates and significant fold change ≥ 2.0 and p-value <0.05 were considered to screen the proteins. Dots in orange, blue and grey represents proteins that are upregulated, downregulated and unchanged respectively. GBC- Gallbladder carcinoma; GSD- Gallstone disease.

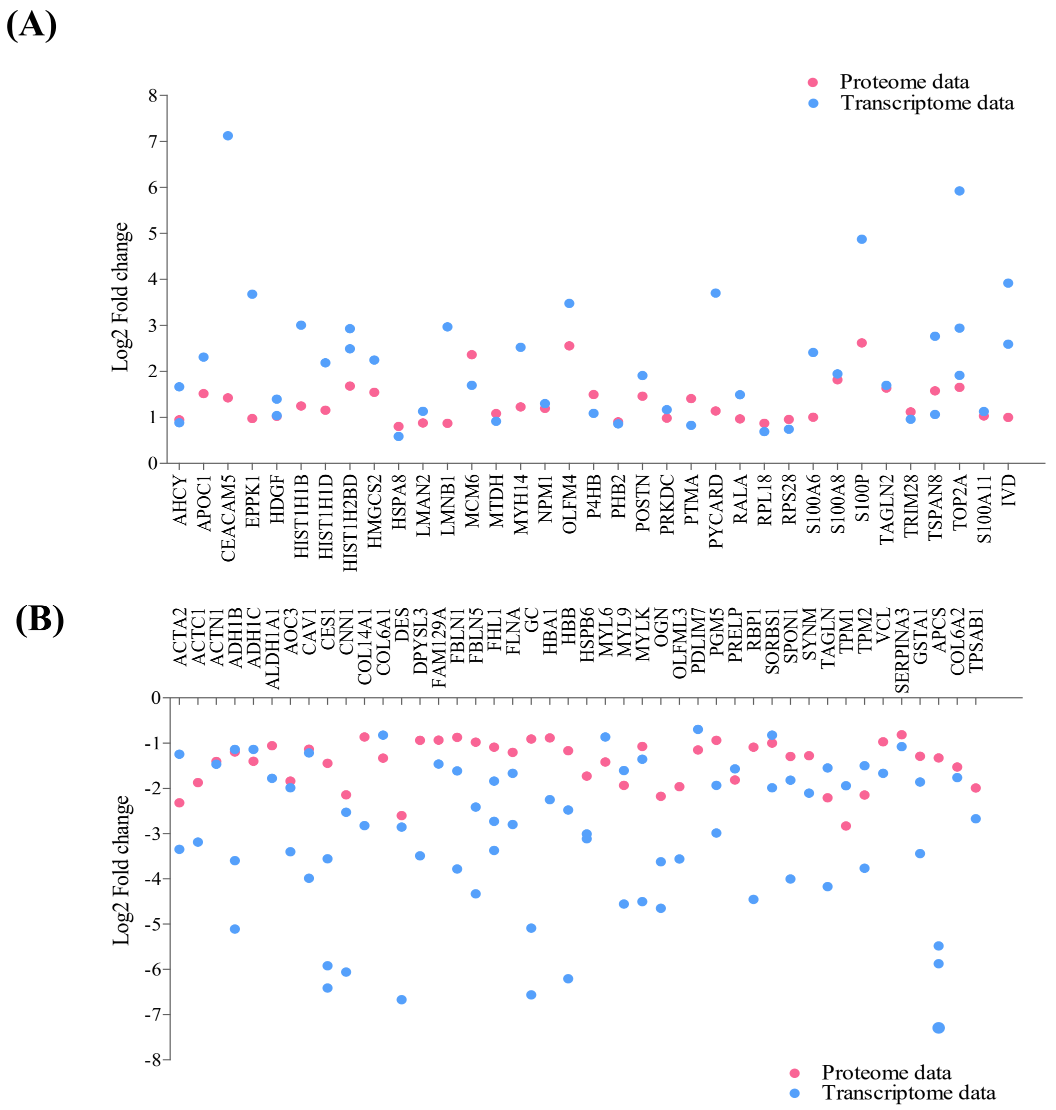

Supplementary Figure S4

Comparison of DEPs in our proteome data with the transcriptome dataset. Proteins showing positive correlation in expression both at protein and transcript level **(A)** Upregulated **(B)** Downregulated. GBC Transcriptome data from published literature was used for the analysis. Log2 fold change in expression level was used for the comparison.

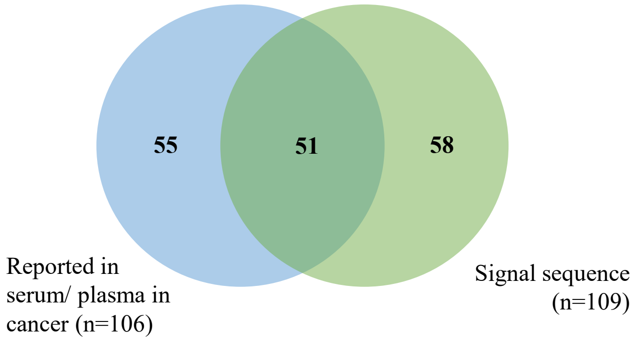

Supplementary Figure S5

Venn diagram showing the DEPs with signal sequence and previously reported to be differentially abundant in serum or plasma in cancer. Signal sequence was predicted for DEPs in early stage GBC using SignalP software version 6.0. Literature search was done to screen the proteins that are reported to be with altered levels in cancer.

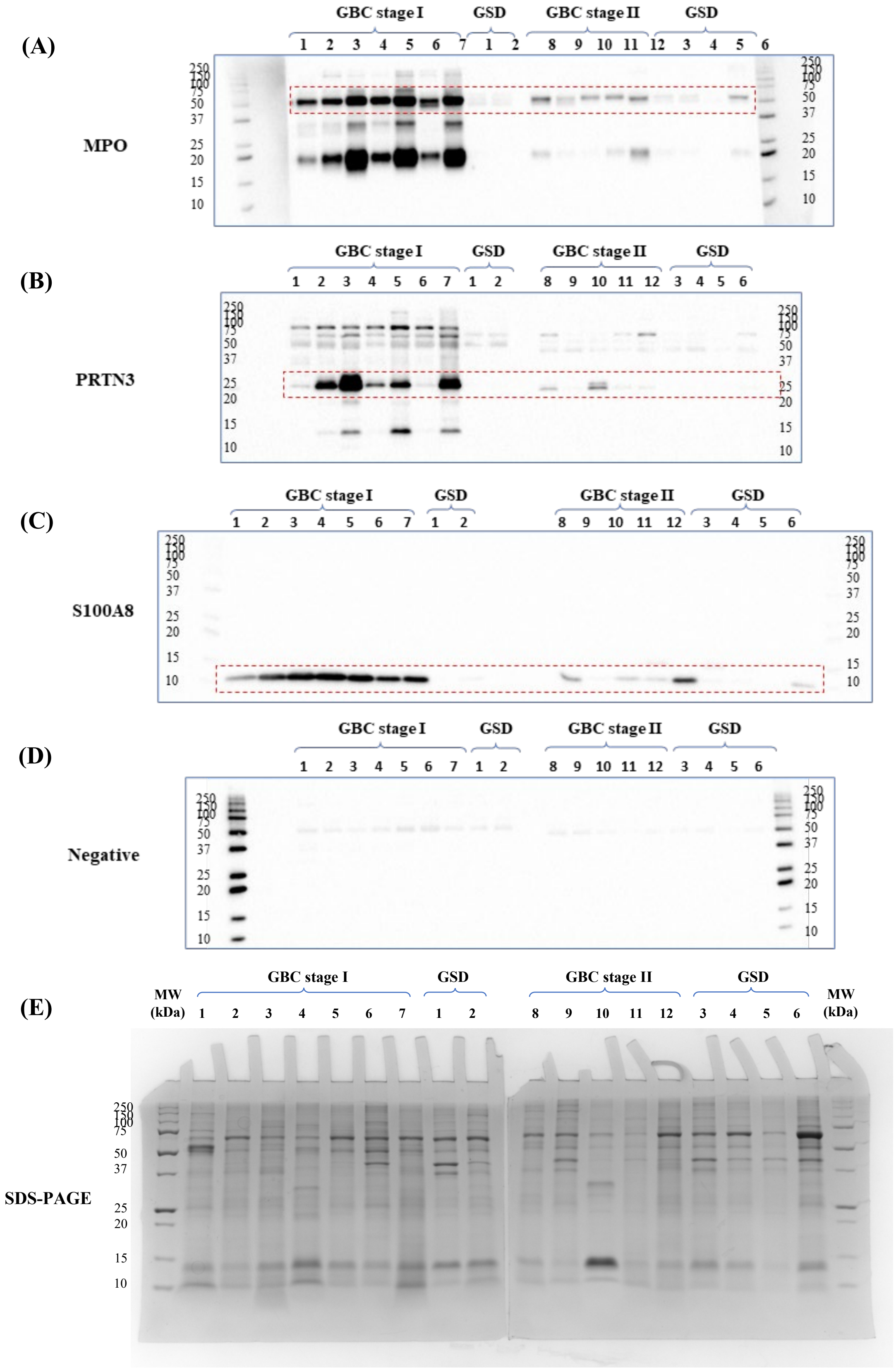

Supplementary Figure S6

Full-length blot images of **Figure 8** for the expression of (A-C) MPO, PRTN3, S100A8 in early stage GBC cases and GSD cases. All GSD lanes were cropped and aligned towards right in the main image. (D) Negative control, the blot without primary antibody, is not presented in the main image. The cropping of the blot images is indicated with red dashed line. (E) SDS-PAGE image showing the profiles of individual samples used in this study. The total density in each lane was used for normalization to ensure equal loading of samples.

MPO

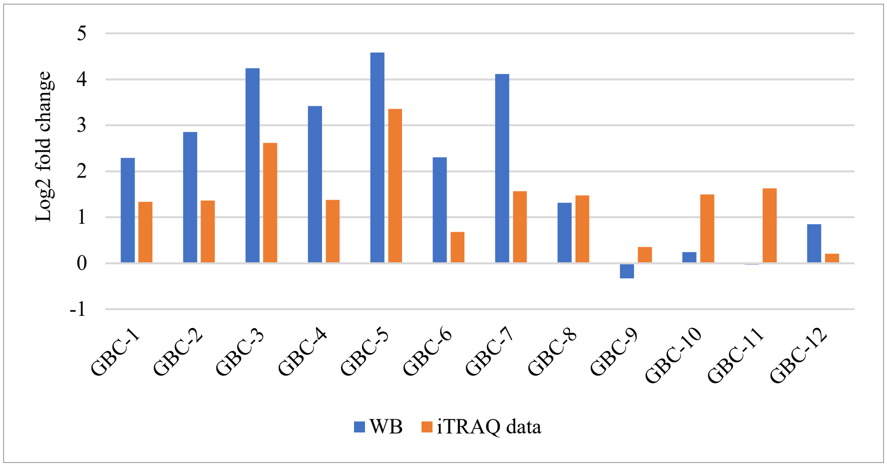

PRTN3

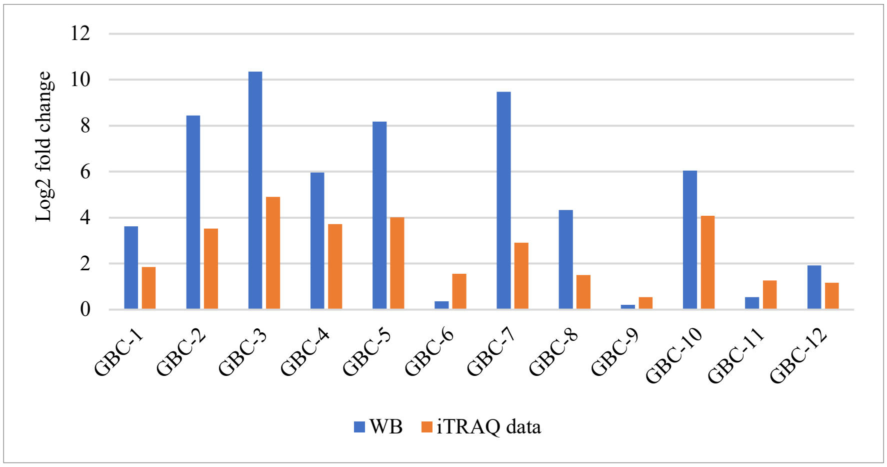

S100A8

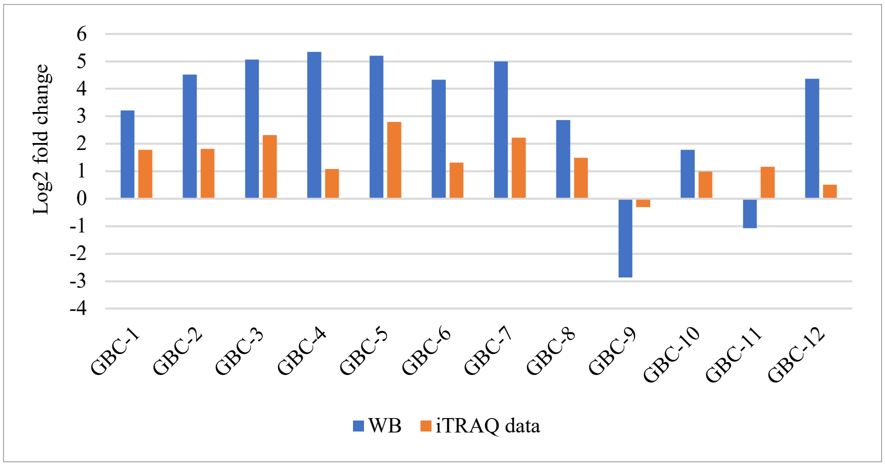

Supplementary Figure S7

Comparative expression of MPO, PRTN3 and S100A8 in GBC cases and controls as observed in quantitative proteomics (iTRAQ data) and Western blot analysis. A positive correlation was found in 91.6% (n=11/12), 100% (n=12/12) and 91.6% (11/12) GBC cases for MPO, PRTN3 and S100A8 respectively among quantitative proteomics and Western blot data. The maximum density of the controls (GSD cases, n=6) was used to derive the fold change. WB- Western blot.

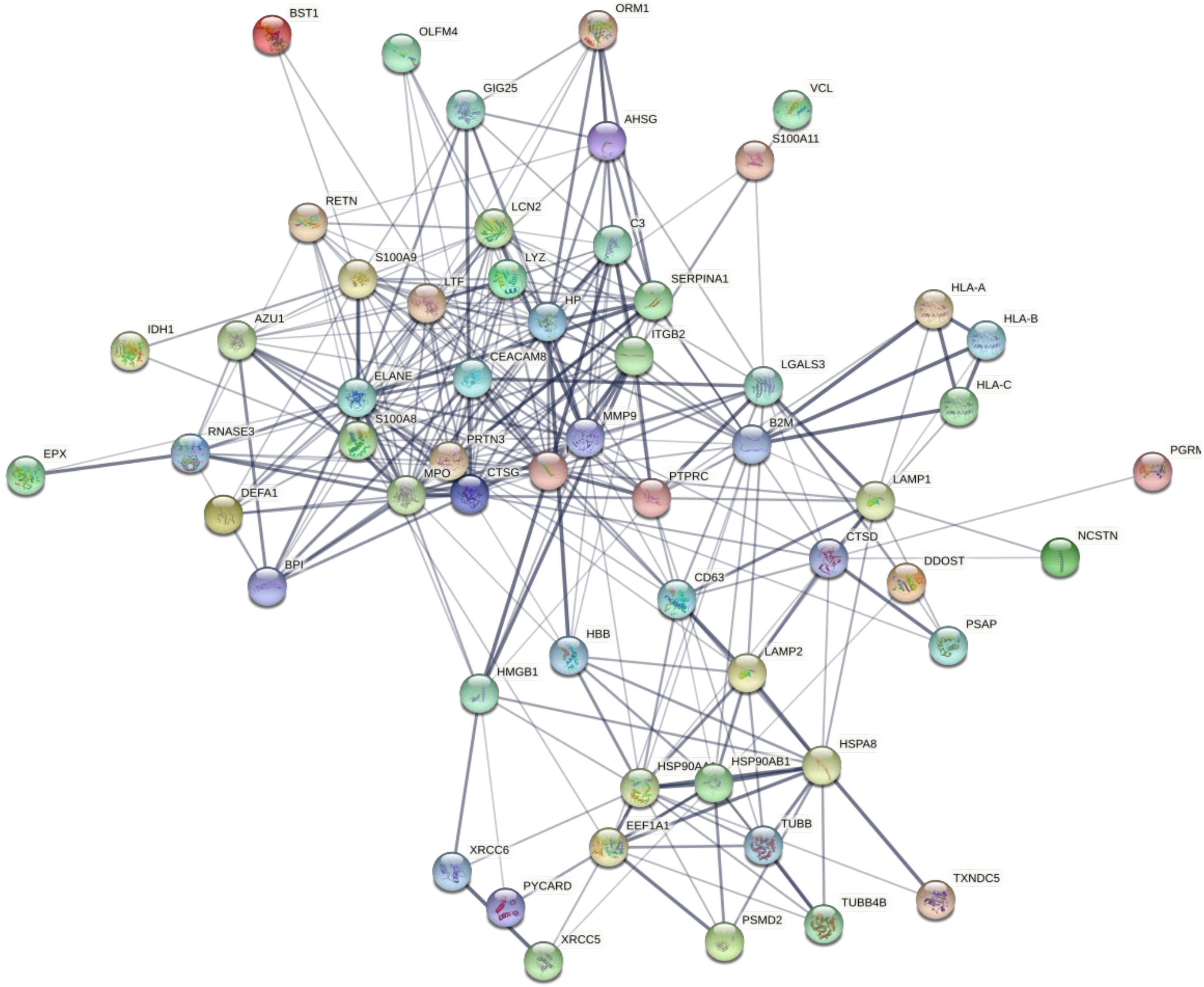

Supplementary Figure S8

Protein-protein interaction network of proteins associated with 'Neutrophil degranulation' pathway. ELANE, ITGAM, MMP9, MPO proteins were the hub molecules.
